# Use of KikGR a photoconvertible green-to-red fluorescent protein for cell labeling and lineage analysis in ES cells and mouse embryos

**DOI:** 10.1186/1471-213X-9-49

**Published:** 2009-09-09

**Authors:** Sonja Nowotschin, Anna-Katerina Hadjantonakis

**Affiliations:** 1 Developmental Biology Program, Sloan-Kettering Institute, New York, NY 10065, USA

## Abstract

**Background:**

The use of genetically-encoded fluorescent proteins has revolutionized the fields of cell and developmental biology and in doing so redefined our understanding of the dynamic morphogenetic processes that shape the embryo. With the advent of more accessible and sophisticated imaging technologies as well as an abundance of fluorescent proteins with different spectral characteristics, the dynamic processes taking place *in situ *in living cells and tissues can now be probed. Photomodulatable fluorescent proteins are one of the emerging classes of genetically-encoded fluorescent proteins.

**Results:**

We have compared PA-GFP, PS-CFP2, Kaede and KikGR four readily available and commonly used photomodulatable fluorescent proteins for use in ES cells and mice. Our results suggest that the green-to-red photoconvertible fluorescent protein, Kikume Green-Red (KikGR), is most suitable for cell labeling and lineage studies in ES cells and mice because it is developmentally neutral, bright and undergoes rapid and complete photoconversion. We have generated transgenic ES cell lines and strains of mice exhibiting robust widespread expression of KikGR. By efficient photoconversion of KikGR we labeled subpopulations of ES cells in culture, and groups of cells within *ex utero *cultured mouse embryos. Red fluorescent photoconverted cells and their progeny could be followed for extended periods of time.

**Conclusion:**

Transgenic ES cells and mice exhibiting widespread readily detectable expression of KikGR are indistinguishable from their wild type counterparts and are amenable to efficient photoconversion. They represent novel tools for non-invasive selective labeling specific cell populations and live imaging cell dynamics and cell fate. Genetically-encoded photomodulatable proteins such as KikGR represent emergent attractive alternatives to commonly used vital dyes, tissue grafts and genetic methods for investigating dynamic behaviors of individual cells, collective cell dynamics and fate mapping applications.

## Background

Cell fate, pattern formation and morphogenesis depend on dynamic cell interactions involving a multitude of cell behaviors and cell populations. One way to gain insight into these events is to label and observe single or groups of cells over time. Several approaches have been established for labeling cells in developing mouse embryos, the mammalian genetically-tractable model of choice, including dye injections, electroporation of nucleic acids or proteins into single or groups of cells [1,2] as well as grafting of genetically-distinct tissues [3,4] or using chimeras [5-7]. Unfortunately, most of these techniques are invasive, and only effective when the tissue or cells of interest are easy accessible for manipulation. Therefore, it has been a challenge to tag single or groups of cells that are not superficially located.

The advent of genetically-encoded fluorescent proteins has afforded the ability to label cells ubiquitously or selectively depending on the *cis*-regulatory elements used in transgene design. Whereas native fluorescent proteins are cytosolic [8], subcellularly-localized fluorescent proteins can be used for higher resolution image information [9]. For example, fluorescent proteins fused to a human histone H2B, since they are bound to active chromatin, allow the visualization and tracking of individual cells within a group [10-12]. Likewise, fluorescent protein fusions that localize to the plasma membrane provide information on membrane dynamics and cell morphology [11,13]. Thus in combination with increasingly sophisticated imaging technology, for example laser scanning confocal or multiphoton microscopy, and on-stage cultures, genetically-encoded fluorescent proteins can provide high resolution information on dynamic cell behaviors.

Binary genetic approaches such as genetically inducible fate mapping (GIFM) based on the Cre/*lox*P system can also be used for cell labeling and fate mapping in mice. GIFM is non-invasive and allows the tagging and tracking of non-superficial cell types [14]. However, cell type specificity relies on availability of *cis*-regulatory elements to drive Cre recombinase transgene expression within specific populations of cells. To date, binary genetic methods have been used to gain information on large groups of cells rather than smaller groups or even individual cells in spatially-defined regions of interest (ROIs). Therefore, methods that combine lineage labeling through transgenesis with spatially-defined ROIs are likely to provide greater flexibility in cell labeling and lineage tracing.

Photomodulatable fluorescent proteins should help overcome the limitations of these approaches by enabling non-invasive selective labeling of cells in ROIs. Various photomodulatable fluorescent proteins including PA-GFP which acquires fluorescence upon activation, PS-CFP2 which changes color from cyan to green fluorescence upon activation, as well as Kaede, KikGR and EosFP which go from a green to a red fluorescence upon activation, have been used to study cell behaviors in different organisms, including *Drosophila*, chick, *Xenopus *and zebrafish [15-19], with preliminary proof-of-principle applications recently reported in mice [20,21]. For example, an alpha-tubulin PA-GFP fusion has used to investigate the dynamics of mesoderm cell migration during Drosophila development [18], and KikGR has been used to study the migratory behaviors of neural crest cells in chick embryos [22].

With so many available photomodulatable proteins, choice of the most suitable for any given application and in any particular organism is not obvious as few direct comparisons have been reported. An extensive comparison of PA-GFP, PS-CFP2, Kaede and KikGR for their applicability in cell migratory behavior and cell lineage analysis in the chick embryo has been reported [23]. Importantly, applications in mice are in their infancy and to date are limited one study in pre-implantation embryos [21] and the characterization of adult transgenic mice. Transgenic strains expressing Kaede have been described in two independent studies [20,24]. One of these studies investigated the movement of cells of the immune system and employed flow cytometry to analyze photoconverted cells [20]. Therefore, live cell imaging experiments using photomodulatable reporters have yet to be fully explored in mice.

Recognizing the need for a comparison of commonly used photomodulatable fluorescent proteins for their prospective use in mice, we compared PA-GFP, PS-CFP2, Kaede and KikGR. We found the green-to-red photoconvertible protein KikGR, engineered from the coral *Favia favus *[25], to be the best suited for cell labeling and fate mapping due to its specific and efficient photoconversion and outstanding brightness compared to the other photomodulatable fluorescent proteins tested in this study. We therefore generated and characterized transgenic mouse strains constitutively expressing KikGR under the *CAG *promoter [26]. We also report the generation of transgenic ES cell lines exhibiting widespread expression of KikGR. We observed bright fluorescence in KikGR transgenic ES cells, embryos and adult mice. We noted rapid and efficient photoconversion of KikGR at various developmental stages. To study cell dynamics in mouse embryos, groups of cells in spatially defined ROIs were photoconverted and followed over time.

This study therefore provides the first comparison of photomodulatable fluorescent proteins for use in ES cells and mice, and reveals the potential utility of these genetically-encoded reporters for investigating individual cell behaviors, population dynamics and cell fate in the mouse embryo.

## Results and Discussion

### Photoconversion of cells and *ex utero *culture of embryos to visualize cell dynamics *in situ*

**Figure 1:**
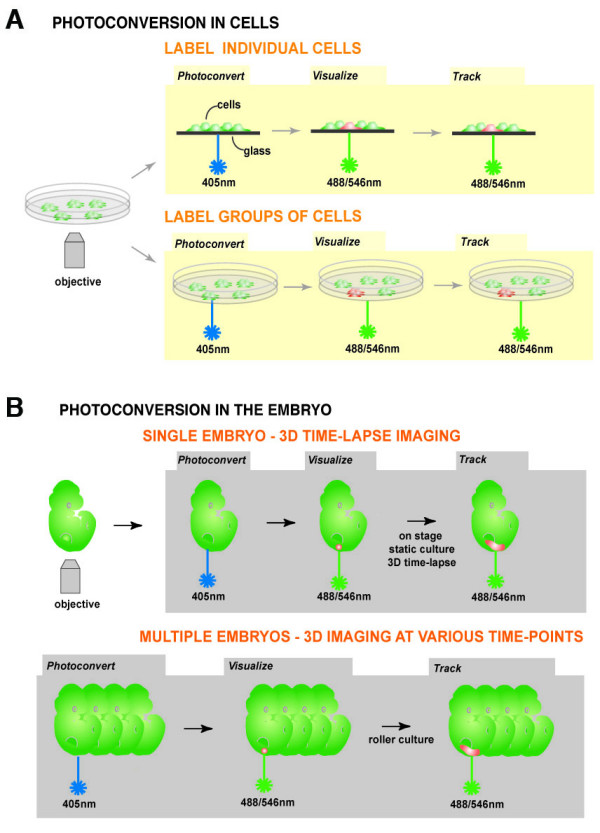
**Schematic representation of the concept of photoconversion of cells in culture and in embryos**. Photoconversion in cells (A): Single cells or group of cells expressing a constitutively active KikGR can be labeled non-invasively. KikGR expressing cells fluoresce green before photoconversion. After exposure to short wavelength light (405 nm) a single or a group of cells of interest can be photoconverted. Photoconverted cells emit red fluorescence and can be tracked over time. Photoconversion in mouse embryos (B): Any KikGR-positive cell or group of cells of interest in a mouse embryo can be photoconverted using short wavelength light (405 nm). Photoconverted red fluorescent cells can be imaged in live static cultured embryos, so that 3D over time (4D) data sets can be acquired. Using live imaging and roller culture, a region of interest (ROI) in multiple embryos (for example all embryos in one litter) can be photoconverted in the same experiment and imaged. This is advantageous for comparing cell behavior in wild type and mutant mice before any visible onset of a phenotype.

Since their spectral properties can be modulated in defined regions of interest, photoconvertible fluorescent proteins provide greater control of labeling specificity over conventional fluorescent proteins. They are therefore superior for precisely and non-invasively labeling single or small cohorts of cells in complex populations (Figure 1A). Our goal was to generate transgenic ES cells and mice with widespread expression of a readily detectable and efficiently photoconvertible fluorescent protein reporter. These would be used for 3D time-lapse imaging cell dynamics *in situ *in ES cell cultures and mouse embryos. Once determined wild type cell behaviors can be contrasted with those resulting from experimental perturbations, for example in mutants. By using photoconversion in combination with *ex utero *embryo culture cells can be tracked over time providing a way to visualize the cell dynamics that direct the emergence of morphologically distinct structures or mutant phenotypes (Figure 1B).

### Construction and evaluation of different photomodulatable proteins in cells

**Figure 2:**
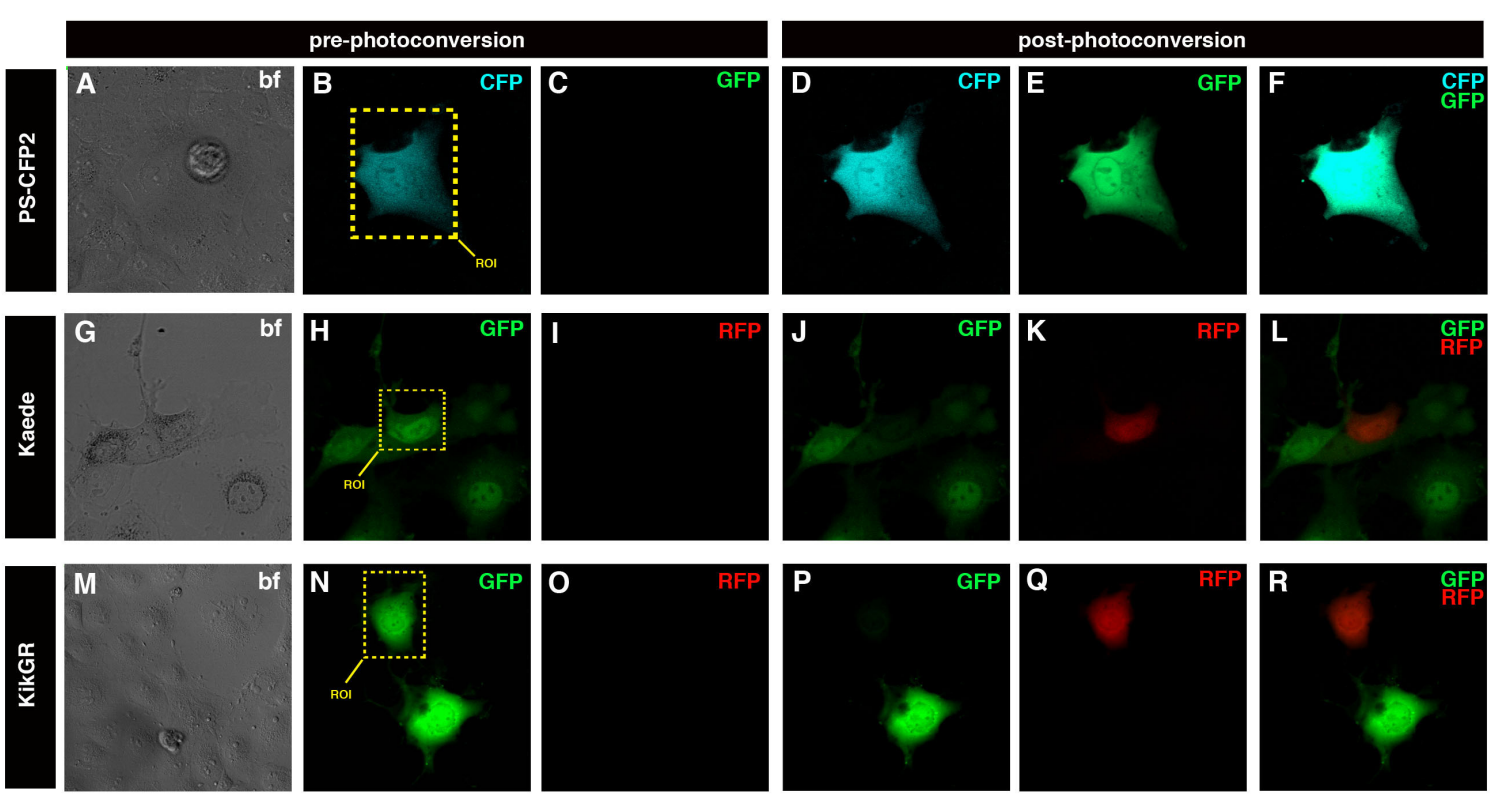
**Comparative photoconversion of PS-CFP2, Kaede and KikGR**. Panels show photoconversion of PS-CFP2 (A-F), Kaede (G-H) and KikGR (M-R) in transient transfected COS7 cells. The ROI that was subjected to short wavelength laser light is depicted in the yellow dashed box. Bright field (BF) images of respective cell transfections (A, G and M). Bright field images are 2D representations (A, G and M). Panels B-F, H-L and N-R show 3D images of converted COS7 cells. Upper row shows photoconversion of PS-CFP2. Cells fluoresce cyan before photoconversion and green after photoconversion. CFP channel (B) and GFP channel (C) before photoconversion. CFP (D) and GFP channel (E) after photoconversion. Merge of the CFP and GFP channel (F). Middle row depicts the photoconversion of Kaede, and bottom row shows the photoconversion of KikGR both of which fluoresce green before, and red after photoconversion. GFP channel (H, N) and RFP channel (I, O) before photoconversion. GFP (J, P) and RFP channel (K, Q) after photoconversion. Merge of the GFP and RFP channels (L, R). Note that you see a stronger CFP signal in D due to signal bleed through of the GFP channel. Note that cells were photoconverted with a 20× objective using 35% laser power of the 25 mW 405 nm laser, however, KikGR was imaged with less gain in the channels of its respective colors as compared to PS-CFP2 and Kaede.

To establish if one of a series of the widely available photomodulatable proteins would be better suited for lineage studies in mouse embryos, we compared PA-GFP [27], PS-CFP2 [28], Kaede [29] and KikGR [25]. All four photomodulatable proteins were cloned under the widespread CAGGS enhancer/promoter, comprising the chicken beta-actin promoter and first exon/intron placed downstream of the CMV immediate early enhancer [26]. COS-7 and ES cells were transfected with the four *CAG *promoter-based plasmids and visualized pre- and post photoactivation. Results are summarized in Table 1. Comparison of expression and photoactivation or photoconversion, respectively, in cells, showed high sensitivity of PA-GFP to UV light and rapid photoactivation. However, even exposure to daylight resulted in instantaneous autoactivation or bleaching of the PA-GFP fluorophore. This was unfavourable since mouse embryos develop *in utero *and must be dissected from maternal tissues using a stereo dissecting microscope. They therefore require illumination of both the microscope stage and animal for removal of the uterus. PS-CFP2 was dim, and its photoconversion was inefficient. Furthermore, given the spectral overlap between the cyan and green forms of the protein, to optimally distinguish photoconverted and unconverted cells linear unmixing of spectra is necessary (Figure 2). Kaede was photoconverted efficiently but was dim compared to KikGR (Figure 2). Kaede also often formed aggregates in COS-7 cells and was toxic in ES cells when expressed at high levels (data not shown). By contrast, photoconversion of KikGR was immediate and very efficient. Furthermore, KikGR did not appear to be toxic when highly expressed in ES cells, and was the brightest of the four evaluated photomodulatable proteins. These features made KikGR the protein of choice for use in ES cells and, by extrapolation, in mouse embryos.

**Table 1:** Comparison of genetically-encoded photomodulatable fluorescent proteins.

*Fluorescent reporter*	*Color pre-activation*	*Color post-activation*	*Comments*
**PA-GFP**	Non-fluorescent	green	Considerable autoactivation (i.e. photoactivation through daylight).
**PS-CFP2**	cyan	green	Dim pre-activation. Inefficient photoconversion. Considerable overlap between pre- and post-activation spectral profiles.
**Kaede**	green	red	Dim. Efficient photoconversion. Aggregates form in COS cells, toxic at when expressed at high levels in ES cells.
**KikGR**	green	red	Bright pre- and post-activation. Efficient photoconversion.

We next established transgenic ES cell lines constitutively expressing *KikGR*. Fluorescent transgenic ES cell colonies were identified by their green fluorescence and picked under an epifluorescence stereo dissecting microscope. Clones were expanded and passaged in 96-well plates, and scored for the maintenance and level of green fluorescent reporter after extended maintenance in culture in the absence of selection. Clones that failed to meet these criteria were discarded from further analysis. The photoconversion and photoefficiency of KikGR was quantified in *CAG::KikGR *transgenic ES cells.

### Photoconversion of KikGR in ES cells

**Figure 3:**
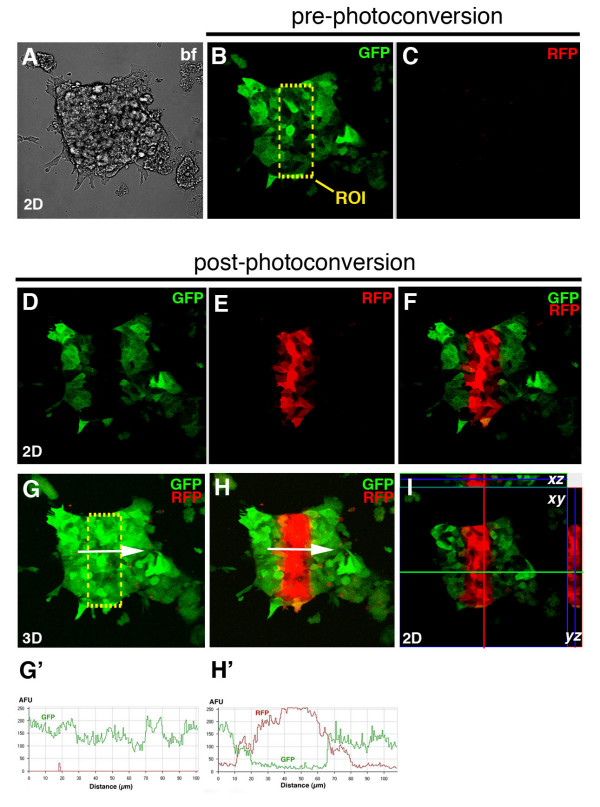
**Photoconversion of widespread expressing *CAG::KikGR *transgenic ES cells**. Panels show 2D (A-F, I) and 3D images (G, H) of the photoconversion of a selected group of cells in an ES cell colony. Bright field image of ES cell colony (A). Image of ES cell colony with green (B) and red (C) channel showing green but no red fluorescence prior to photoconversion. Yellow dashed box show regions of interest (ROI) that is to be photoconverted. After photoconversion, cells in an ROI do not fluoresce green (D) but red (E). Merge of green and red channel after photoconversion, showing complete photoconversion of cells in the region of interest. 3D image of ES cell colony before photoconversion. All cells emit green fluorescence (G). Line plot showing average fluorescent intensity measured along the white arrow in G (G'). 3D image of ES cell colony after photoconversion. Photoconverted cells emit red fluorescence (H). Line plot showing average fluorescent intensity measured along the white arrow in H showing complete photoconversion of cells, i.e. shift of emission spectrum from green to red in photoconverted region (H'). 2D orthogonal representation of photoconverted ES cell colony showing complete photoconversion in the region of interest through entire *z*-stack (I).

**Figure 4:**
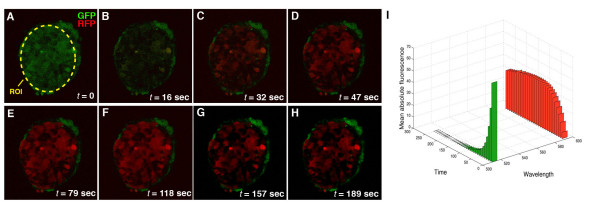
**Efficiency of KikGR photoconversion over time with constant exposure to short wavelength laser light**. Continuous scanning over time with 405 nm laser light excitation (30% laser power; 25.0 mW) of an ES cell colony, constitutively expressing KikGR, shows fast and complete photoconversion. Fluorescence in green and red channels at different times of 405 nm exposure (A-H). Yellow dashed ellipse depicts region of interest (ROI) exposed to 405 nm laser light. Plot of time series in A-H shows shift in emission spectra from 513 nm to 596 nm (I).

Imaging of photoconversion in an ES cell colony constitutively expressing KikGR using short wavelength laser light (405 nm) revealed complete and fast photoconversion of KikGR from green to red fluorescence (Figures 3 and 4). Any ROI could be defined and efficiently photoconverted without compromising cell viability. Complete photoconversion was demonstrated by imaging *z*-stacks of photoconverted ES cells (Figure 3A-F). 2D orthogonal representation of *z*-stack of ES cell colony shows that only photoconverted cells with no residual green fluorescence reside within the square ROI in sections taken throughout the *z*-stack (Figure 3I). Line plots illustrating the average fluorescent intensity along a line plotted across an ROI before and after photoconversion demonstrated a complete shift of emission from green to red fluorescence (Figure 3G, G' H, H').

Exposure of a circular ROI defined within in an ES cell colony to 405 nm light (30% laser power, 25.0 mW) demonstrated fast photoconversion of KikGR (Figure 4A-E). Complete photoconversion was reached in 79 seconds under the experimental conditions used. Continued exposure of photoconverted cells eventually lead to photobleaching of the fluorophore and a slight reduction in red fluorescence (Figure 4G-H). Fluorescent intensities of the green and red channel plotted over time illustrated the rapid shift in the emission spectrum from 516 nm to 593 nm after exposure to 405 nm light (Figure 4I).

### Expression and function of KikGR in adult transgenic mice and post-implantation embryos

**Figure 5:**
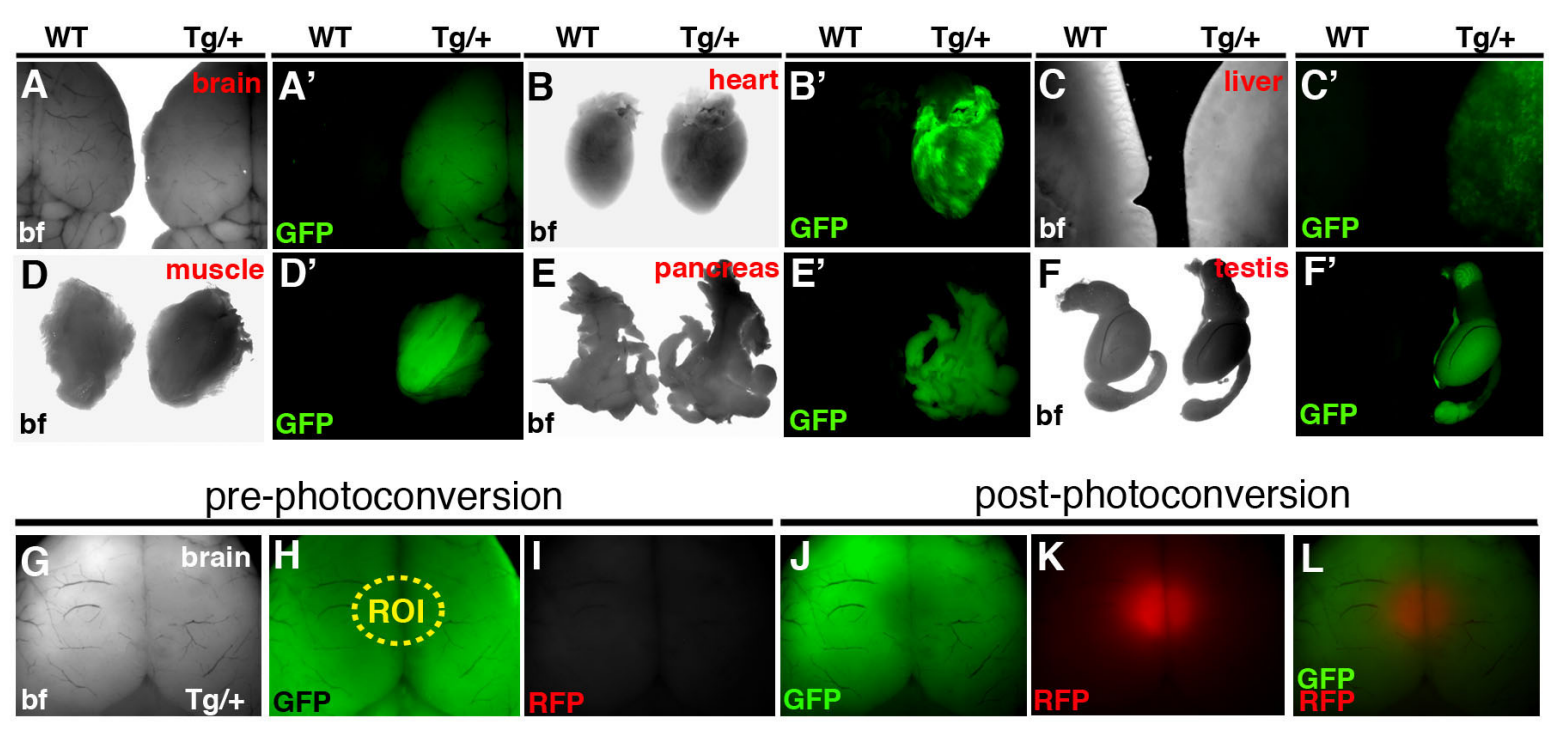
**Widespread KikGR expression in organs of *CAG::KikGR*^*Tg*/+ ^adult mice**. Panels of bright field and corresponding dark field epifluorescent images of organs taken from a 6 week old *CAG::KikGR*^*Tg*/+ ^mouse and a non-transgenic littermate. Brain (A, A'), heart (B, B'), liver (C, C'), muscle (D, D'), pancreas (E, E') and testis (F, F'). Panels of bright field and corresponding dark field epifluorescent images (green and red channels) of the brain before and after photoconversion using a UV filter (G-L). Yellow dashed ellipse depicts region of interest (ROI) to be photoconverted (H). Green channel (H, J). Red channel (I, K). Overlay of images of green and red channel (L).

**Figure 6:**
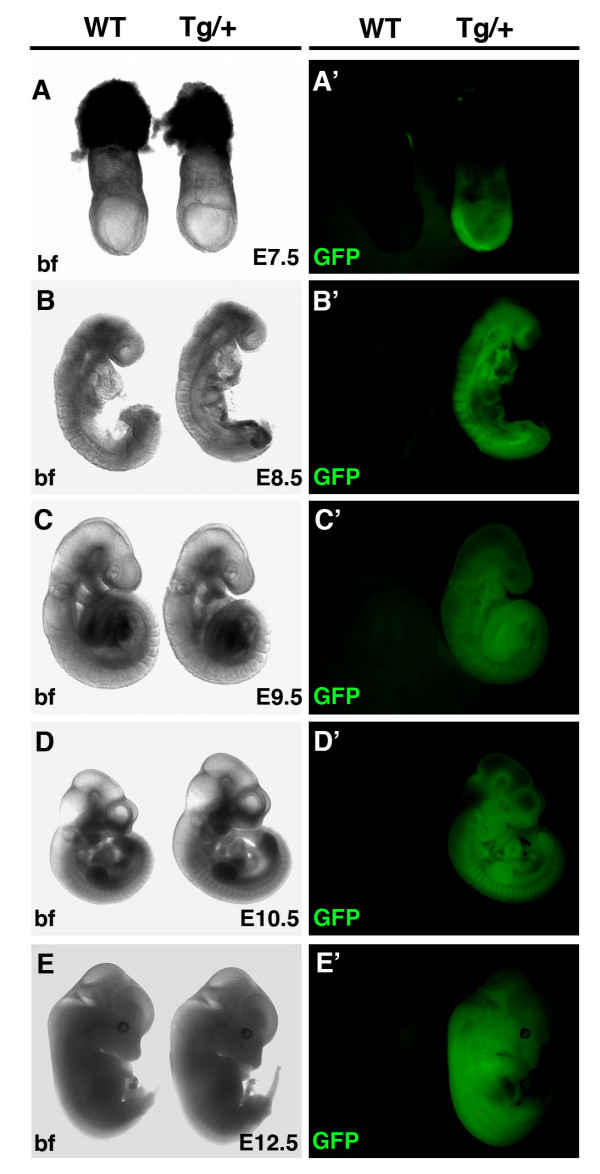
**Widespread KikGR expression in *CAG::KikGR*^*Tg*/+ ^embryos**. Panels of bright field and corresponding dark field epifluorescent images of *CAG::KikGR*^*Tg*/+ ^embryos and non-transgenic littermates at E7.5 (A, A'), E8.5 (B, B'), E9.5 (C, C'), E10.5 (D, D') and E12.5 (E, E').

The *CAG::KikGR *construct was used to generate transgenic strains of mice with widespread expression of KikGR. Three founders were characterized. Each line exhibited widespread expression, however, all data presented in this article derive from a single line. To characterize the *CAG::KikGR *mouse strains and confirm that they exhibited widespread transgene expression as detected by green fluorescence, adult organs and post-implantation embryos of various stages were imaged using wide field epifluorescent microscopy (Figures 5 and 6). Dissection of organs from a 6 week old adult *CAG::KikGR*^*Tg*/+ ^animal, and its wild type littermate revealed robust widespread expression of KikGR in organs of the hemizygous transgenic (Figure 5A-F). In addition, embryos of post-implantation stages ranging from embryonic (E) day 7.5 through to E12.5 exhibited strong widespread green fluorescence (Figure 6). Photoconversion of an ROI in the brain of an *CAG::KikGR*^*Tg*/+ ^using a UV filter and epifluorescent illumination demonstrated the functionality of the KikGR protein in the transgenic mouse (Figure 5G-L). Importantly, *CAG::KikGR*^*Tg*/+ ^animals were indistinguishable from their littermates, both as embryos and adults, suggesting that readily detectable levels of KikGR expression are developmentally neutral and non-toxic.

### Live imaging population dynamics using KikGR

Coordinated behaviors within cell populations are integral to a wide variety of morphogenetic events. Using transgenesis to direct KikGR expression in embryos and photoconversion of ROIs in combination with 3D time-lapse imaging and *ex utero *embryo culture, the collective cell behaviors directing the morphogenesis of the mouse embryo can be probed. For example, given the precision of cell labeling using defined ROIs, it can be determined if collective cell behaviors such as convergence and extension occur in mouse embryos. A convergence and extension type cell behavior can be investigated through photoconversion of cells contained in a rectangular ROI, and measurement of the height-to-width over time. One can then determine if the starting ratio is maintained or skews in any particular dimension over time, with the latter trend suggestive of a convergent extension type cell behavior.

**Figure 7:**
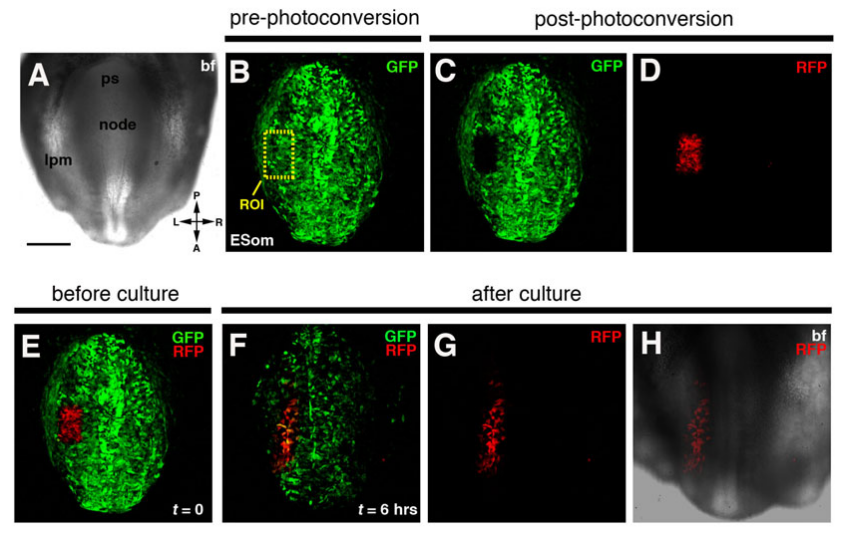
**Photoconversion of cells in the lateral plate mesoderm (lpm) of an E8.0 *CAG::KikGR*^*Tg*/+ ^embryo: live imaging of behaviors of cell cohorts**. Panels show 2D (bright field) and 3D (green and red channels) laser scanning confocal images of a posterior view of an E8.0 *CAG::KikGR*^*Tg*/+ ^embryo (A-H). Bright field image (A). KikGR expression in an E8.0 embryo prior to photoconversion. Yellow dashed rectangle, region of interest (ROI), demarcates population of cells to be excited with laser light of 405 nm wavelength (B). Loss of green fluorescence in boxed ROI after photoconversion (C). Cells in boxed ROI fluoresce red after photoconversion (D). Merge of red and green channels after photoconversion showing labeled lpm cells at start of embryo culture (E). Panels show migration of photoconverted cells in lpm of embryo after 6 hrs. Dimensions of cell population photoconverted have become longer and thinner (F-G). Merge of green and red channel (F). Red channel (G). Overlay of red channel onto bright field image (H). ESom, early somite stage; ps, primitive streak. Scale bar indicates 200 μm.

Figure 7 shows a posterior-ventral view of an E8.5 (~6 somite stage) embryo. By photoconversion of an ROI of known dimensions within the caudal part of the unsegmented (presomitic) paraxial mesoderm (Figure 7A-D), and subsequent imaging after a 6-hour period of roller culture, the dimensions (height-to-width ratio) of the photoconverted cell population were seen to change over time (Figure 7E-H). This change in the height-to-width ratio (N = 3 embryos) suggests that cells of the unsegmented paraxial mesoderm of a 6-somite stage mouse embryo may undergo a convergent extension type collective cell behavior, an observation that warrants further investigation.

### Live imaging of cell fate in the mouse embryo

**Figure 8:**
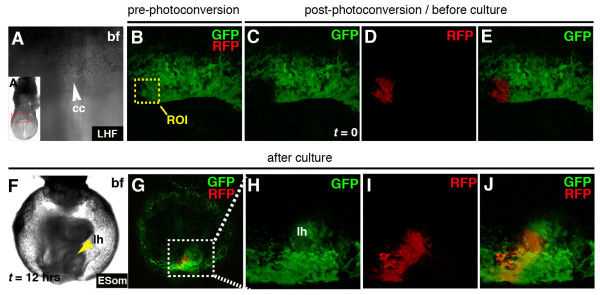
**Live imaging cell fate during mouse cardiac morphogenesis**. Panels show 2D (bright field) and 3D (green and red channels) laser scanning confocal images of photoconversion cells on the right side (ROI) of the cardiac crescent (cc) of an E7.5 *CAG::KikGR*^*Tg*/+ ^embryo (A-E). After 12 hrs of roller culture the embryo has formed a linear heart tube (lh) and photoconverted cells can be detected on the right side of the linear heart tube (F-J). Widefield image of an E7.5 embryo. Red dashed box shows region of cc depicted in panels A-E (A'). Bright field image of cardiac crescent before culture (A) and embryo after culture (F). Red and green channel (B, E, G and J). Green channel (C and H). Red channel (D and I). White dashed box in panel G shows region depicted in panels H-J.

Next, to determine if *CAG::KikGR *transgenic mice were suitable for following cell fates, we investigated the first steps during cardiac morphogenesis (Figure 8). A small group of (<30) cells residing on the right side of the cardiac crescent of an E8.0 embryo were photoconverted (Figure 8A-E). After 12 hrs in roller culture, during which time cells of the cardiac crescent fuse at the midline and go on to form a linear heart tube, the photoconverted cells and/or their progeny became incorporated in, and restricted to, the right side of the linear heart tube (Figure 8F-G). These results reveal that cells located on the right side of the cardiac crescent only contribute to the right side of the linear heart tube [30]. These data demonstrate that photomodulatable fluorescent protein reporters should facilitate precise labeling and fate mapping of defined cell populations during mouse embryonic development.

### Tracking cells over time using 3D time-lapse imaging and on-stage mouse embryo culture

**Figure 9:**
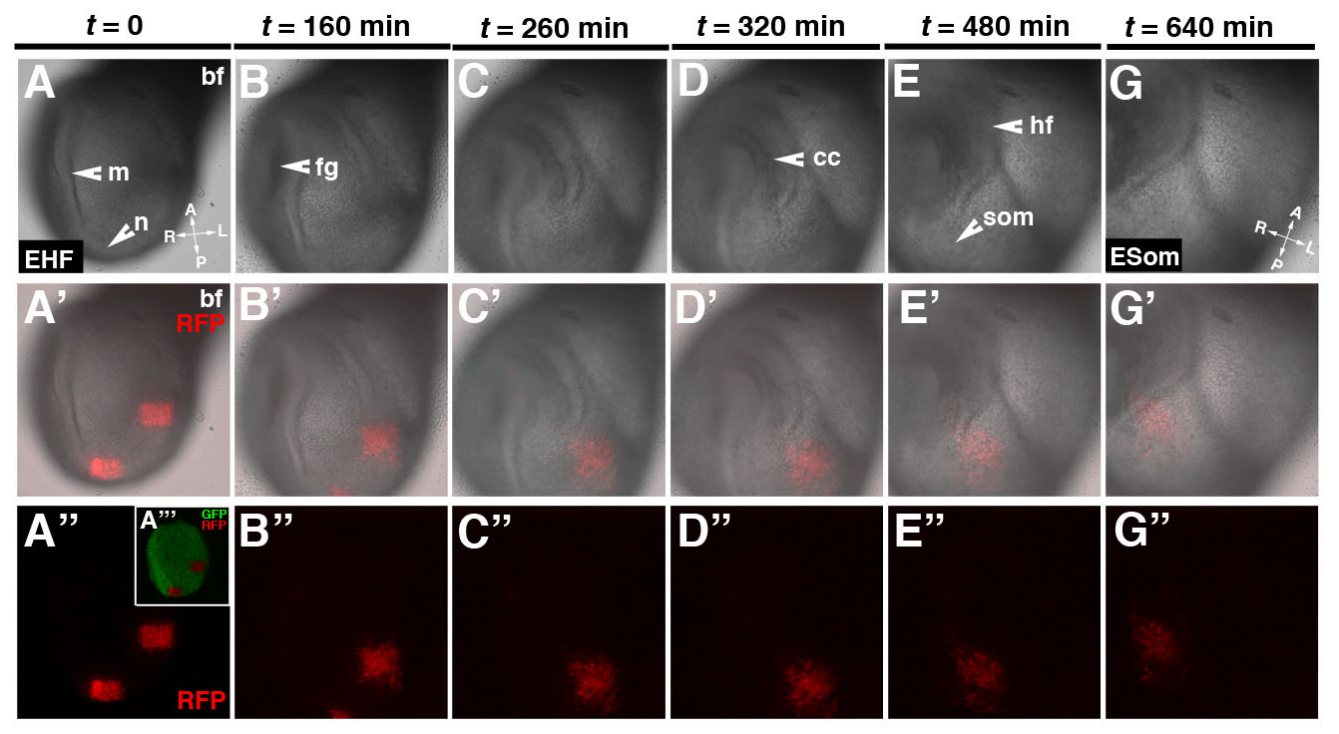
**Live imaging photoconverted KikGR-positive cells in a mouse embryo: photoconversion, *ex utero *culture and 3D time-lapse laser scanning confocal imaging do not affect embryonic development**. Panels show sequence 3D stills over a time of 640 min of a *CAG::KikGR*^*Tg*/+ ^embryo developing from an early head fold stage to an early somite stage (A-G). The series depicts the developing cardiac crescent (cc), headfolds (hf) and condensing somites (som). Two regions of photoconverted cells are shown over time. Cells of photoconverted region located in the left side of the embryo migrate anteriorly into the cardiac crescent. Bright field images (A-G). Merge of RFP channel and bright field (A'-G'). RFP channel (A"-G"). GFP and RFP channel (A"'). m, midline; n, node.

Ultimately, time-lapse image acquisition provides the highest resolution information on cell dynamics. Therefore, we next assessed if we could photoconvert a group of cells and track them *in situ *in embryos that were cultured on a microscope stage as opposed to in a roller culture incubator [31]. Our data suggest that, as with roller culture, on-stage static embryo culture as well as multiple exposures to laser light at timed intervals did not adversely affect embryonic development. Still frames of the bright field channel of a 3D time-lapse imaging experiment show development of a mouse embryo cultured static from the early headfold stage (EHF - E7.75) to the early somite stage (ESom) that is equivalent in timing and morphology to wild type *in utero *development (Figure 9). The image sequence depicts co-ordinate morphogenetic events including the invagination of the foregut pocket, development of the headfolds and condensation of the rostral somites. The group of photoconverted cells retains red fluorescence and can be followed throughout the 10-hour time-lapse and culture period. These data reveal the dynamics and expansion of a population of mesodermal cells migrating into the cardiac crescent (see Additional file 1).

## Conclusion

We have compared four widely available photomodulatable fluorescent proteins: PA-GFP which photoactivates to produce green fluorescence, the cyan-to-green PS-CFP2, and Kaede and KikGR, two green-to-red photoconvertible fluorescent proteins. Our data demonstrate that the photomodulatable fluorescent protein KikGR is suitable for a number of live imaging applications in ES cells and in mouse embryos. KikGR is developmentally-neutral, does not autoactivate after exposure to broad spectrum white light, undergoes rapid and complete photoconversion when exposed to light of a specific wavelength, and exhibits bright fluorescence before and after photoconversion.

*CAG::KikGR *transgenic mice lend themselves to a variety of live imaging applications for investigating individual cell behaviors, population dynamics and cell fate in the mouse embryo. Defined ROIs permit high temporal and spatial specificity for cell labeling, yielding high-resolution information on cell dynamics *in vivo*. Having established widespread expressing KikGR strains and demonstrated their utility, the development of strains exhibiting lineage-specific KikGR should in the future should provide greater spatial specificity for cell labeling. Moreover, the future development of mouse strains expressing subcellularly-localized KikGR or monomeric KikGR fusion proteins should provide greater spatial resolution for live imaging.

## Methods

### Plasmids and constructs

*CAG::KikGR *was generated by PCR of KikGR from pKIkGR1-S1 (MBL), (GenBank Accession number: AB193293) [25] using oligos EcoRI-KikGR_5': GTTACCGGAATTCCGGATGGTGAGTGTGATTACATCAGAA and EcoRI-KikGR_3': CAATCCGGAATTCCGGTTACTTGGCCAGCCTTGGCAGCCCGGA. The resulting PCR product was cut with EcoRI cloned into pCAGGS [26]. *CAG::PA-GFP *was generated by PCR of *PA-GFP-N1 *[27] using oligos EcoRI-PAGFP_5': GTTACCGGAATTCCGGATGGTGAGCAAGGGCGAGGAGCTG and EcoRI-PAGFP_3': CAATCCGGAATTCCGGTTATCTAGATCCGGTGGATCCCGG. *CAG::PSCFP2 *was generated by PCR of PS-CFP2 from *pPS-CFP2-N *(Evrogen) [32] using oligos EcoRI-PSCFP2_5': GTTACCGGAATTCCGGATGAGCAAGGGCGCCGAGCTGTTC and EcoRI-PSCFP2_3': CAATCCGGAATTCCGGTTACTTGTACAGCTCATCCATGCC. The resulting PCR products for each vector were cut with *Eco*RI and cloned into *p*CAGGS [26]. *CAG::Kaede *was generated by PCR of Kaede from pCS2-Kaede [29] using oligos Kaede-5F-EcoR1: CCGGAATTCCGG ATGGTGAGTCTGATTAAACCAGAAATGAAG and Kaede-3R_EcoR1: CCGGAATTCCGGTTACTTGACGTTGTCCGGCAATCCAGAATG. The resulting PCR products for each vector were cut with *Eco*RI and cloned into *p*CAGGS [26].

### Generation of transgenic ES cells

All constructs were tested for fluorescence in COS7 cells and R1 ES cells [33]. R1 ES cells were maintained under standard conditions [31]. Transgenic ES cell lines constitutively expressing *CAG::KikGR *were generated by co-electroporation of *Sal*I linearized *CAG::KikGR *construct and a circular *PGK-Puro-pA *plasmid conferring transient puromycin resistance. Puromycin selection was carried out exactly as described previously [8].

### Mouse breeding

*CAG::KikGR *was linearized with *Sal*I and gel-purified using routine protocols [31] DNA was injected into C57BL/6 zygotes at the Memorial Sloan-Kettering Cancer Center Transgenic Core Facility. F0 founders were screened by PCR and expression of green fluorescence, resulting in 3 founder lines. Founders were mated to ICR females to recover embryos and subsequent F1 generation adults.

### Embryo collection and culture

Post-implantation embryos and organs were dissected in DMEM/F12 containing 5% fetal calf serum and cultured in media comprising 50% rat serum, 50% DMEM/F12 supplemented with 1% L-glutamine and 1% Penicillin/Streptomycin.

### Image acquisition

All images of mouse embryos or organs presented in the figures are of living embryos or freshly dissected (unfixed) tissues maintained under physiological conditions. ES cells were also imaged live. Wide-field images were acquired on a Leica MZFLIII stereo dissecting microscope or Zeiss Axiovert 200 M inverted microscope equipped with epifluorescent illumination using appropriate filter sets. Laser scanning confocal data was acquired using a Zeiss LSM510 META scan head fitted onto a Zeiss Axiovert 200 M. Fluorophores were excited with a 488 nm Argon laser line (green) and a 543 nm HeNe laser (red). Objectives used were a plan-apochromat 20×/0.75 and a plan-apochromat 10×/0.45. Confocal images were acquired as sequential optical *x*-*y *sections taken at 1-2 μm *z *intervals.

### Photoconversion

COS-7 cell expressing PS-CFP2, KikGR and Kaede were visualized under standard conditions for visualization of CFP and GFP, respectively using a 458 Argon laser (45%) and a 488 nm Argon laser (5% power), respectively, with a plan-apochromat 20×/0.75 objective. A 405 nm Diode laser (set at 35% power) was used for photoconversion of COS7 cells in culture with 1× 50 iterations. Photoconverted KikGR and Kaede were imaged using a 543 nm HeNe laser (80% power) for red fluorescence excitation. Both photoconverted and unconverted KikGR and Kaede were imaged using 488 nm Ag laser (5% power) for green fluorescence and 543 nm HeNe laser (80% power) for red fluorescence in multi-track mode. Photoconverted PS-CFP2 was imaged using a 458/488 nm Argon laser (45%/5% laser power) for cyan and green fluorescence respectively, in multi-track mode. Cells and embryos expressing KikGR were visualized under standard conditions for visualization of GFP, using a 488 nm Argon laser (5% power) with a plan-apochromat 20×/0.75 objective. A 405 nm Diode laser (set at 100% power) was used for photoconversion of ES cells in culture with 1 × 50 iterations, and cells *in vivo *in mouse embryos with 1 × 75 iterations. Photoconverted KikGR was imaged using a 543 nm HeNe laser (80% power) for red fluorescence excitation. Both photoconverted and unconverted KikGR were imaged using 488 nm Ag laser (5% power) for green fluorescence and 543 nm HeNe laser (80% power) for red fluorescence in multi-track mode. For time-lapse imaging after photoconversion, 488 nm and 543 nm lasers (3% and 50% power, respectively) excitation, were used with plan-apochromat 10×/0.45 or plan-apochromat 20×/0.75 objectives capturing 512 × 512 pixels per frame. For quantifying the photoefficiency of KikGR continuous exposure at 405 nm (30% laser power; 25.0 mW) was used.

### Image processing

Raw data was processed using Zeiss LSM software (Carl Zeiss Microsystems at http://www.zeiss.com/) and Adobe Photoshop CS2 (Adobe Systems, Inc., San Jose). Re-animation of data to generate movies of time-lapses was performed using QuickTime Pro (Apple Computer, Inc at http://www.apple.com/quicktime/).

## Authors' contributions

SN - conceived, designed and carried out the experiments, analyzed the results and drafted the manuscript. AKH - conceived, funded and supervised the project. Both authors critically read and revised the manuscript, and approved the final version.

## Supplementary Material

Additional file 1 **Live imaging and photoconversion of KikGR, a green-to-red fluorescent protein, in a mouse embryo**. 3D time-lapse of an E7.5 (early headfold stage - EHF) *CAG::KikGR *embryo from the EHF - ESom (early somite stage). KikGR is a green-to-red photoconvertible fluorescent protein. In the *CAG::KikGR *strain all cells express KikGR. There are three consecutive sequences in the movie. The first depicts the bright field channel alone to illustrate normal development (foregut invagination, development of the headfolds as well as somite condensation), second the red fluorescent channel (post conversion) overlayed on bright field, and third the red fluorescent channel. Two square regions of interest (ROIs) have been converted within the mesoderm. Each frame depicting fluorescence represents a 3D reconstruction of a z-stack (266 μm). The interval between slices is 7 μm. The time interval between z-stacks is 20 min. The time-lapse sequence covers 9 hours and 20 min. The embryo is suspended from its ectoplacental cone using a human eyelash. Anterior of the embryo is to the top left. Click here for file
